# Lanthanide Complexes of Substituted **β**-Diketone Hydrazone Derivatives: Synthesis, Characterization, and Biological Activities

**DOI:** 10.1155/2011/531946

**Published:** 2011-06-22

**Authors:** W. H. Hegazy, I. H. Al-Motawaa

**Affiliations:** ^1^Chemistry Department, College of Science, King Faisal University, Al-Ahsa 31982, Saudi Arabia; ^2^College of Science, Suez Canal University, Suez, Egypt

## Abstract

A series of *β*-diketone hydrazone derivatives have been synthesized through condensation of *β*-diketone with aromatic aldehydes followed by reaction with phenylhydrazine. The structure of the ligands and intermediates are well defined through elemental and spectroscopic analyses. These hydrazones are potential ligands toward lanthanide metal ions. New complexes of trivalent Scandium, Yttrium, Lanthanum, and Cerium have been synthesized. The composition of these complexes is discussed on the basis of elemental analyses, IR, magnetic moments, and thermal analyses. The prepared complexes were screened for antibacterial and antifungal properties and have exhibited potential activity.

## 1. Introduction

Metal complexes of *Schiff* bases occupy a central role in coordination chemistry for analytical, physical, and biochemical purposes [1–19]. Many complexation process of **β**-diketones *Schiff* bases and/or hydrazones with lanthanide metal ions have been synthesized and characterized by elemental analysis, DTA-TG,X-ray, IR, fluorescence, UV spectra,and molar conductance [20–31].Biochemical activities of some complexes were reported [15].

The present investigation deals with the preparation of some new **β**-diketone dihydrazone derivatives (*L^1^–L^12^*) and their mononuclear Sc(III), Y(III), La(III), and Ce(III) complexes. The prepared ligands were characterized by elemental analysis, IR, ^1^H NMR, and mass spectra. Elemental analysis, IR, magnetic susceptibility measurements, TG, and conductivity measurements characterize the prepared complexes. The antibacterial activities of the prepared complexes were assessed against Gram-positive bacteria *B. subtilis* and *S. aureus* and Gram-negative bacteria *E. coli* and *S. typhi*. Prepared complexes were also screened for their antifungal activities against two fungi (*A. niger* and * C. albicans*).

## 2. Results and Discussion

**Figure 1:**
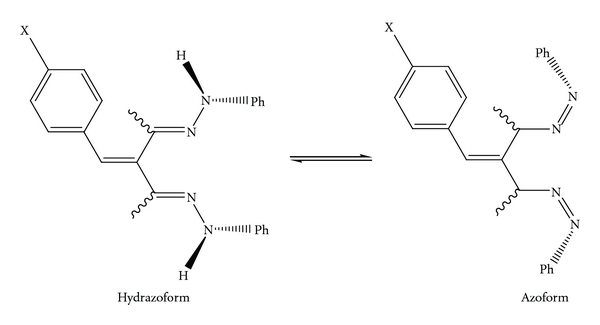
Tautomerization of the ligands.

The synthesized ligands *L^1^–L^12^* were characterized by their physical properties, elemental analyses, IR Spectra, ^1^H NMR, and mass spectra. The data are given in Tables 1–4, respectively.  Figure 1 represents tautomerization of the ligands. 

The elemental analyses of the ligands, Table 1, were found in good agreement with the calculated data (±0.9%).

The infrared spectra of the ligands show broad bands in the region 3449.46–3208.93 cm^−1^ assigned to N–H stretching vibrations, strong-intensity bands in the region 1601.2–1594.04 cm^−1^ assigned to C=N stretching vibrations, strong-intensity bands in the region 1502.21–1442.31 cm^−1^ assigned to interaction (coupling) between C–N stretching and N–H bending vibrations of the C–H–N group, and medium-intensity bands in the region 1376.13–1320.88 cm^−1^ assigned to N=N asymmetric stretching vibrations of the azoform of the ligands which formed as a result of the azo-hydrazo tautomerism [21]. Substitutions at the ortho- and parapositions enhance the mesomeric effect, which activate azo-hydrazo tautomerism. Weak intensity bands in the region 1281.66–1182.83 cm^−1^ are assigned to N–H inplane bending vibrations. Medium intensity bands in the region 1109.24–1060.85 cm^−1^ are assigned to N–N stretching vibrations. It is notable that no bands around 1700 cm^−1^ are observed which confirms the condensation of C=O groups of the Knövenagel condensation with phenylhydrazine [31, 32]. Two medium-intensity bands corresponding to C=C stretching vibrations of the aromatic rings are shown around 1600 and 1500 cm^−1^. Selected infrared data of the new ligands are listed in Table 2.

The ^1^H NMR chemical shifts and coupling constants of DMSO-d_6_ are given in Table 3 suggesting the existence of two tautomeric forms except for ligands *L_9_–L_12_*. Two sets of signals are observed for the methyl group for ligands *L_1_–L_8_*. The resonance of the CH groups of the azoform is readily detectable for all ligands, whereas the peaks of the two NH groups of the hydrazoform (*L_1_–L_8_*) are broad in the case of *L_1_–L_5_*. The above IR and NMR spectroscopic features point to an azo-hydrazo tautomerism for ligands *L_1_–L_8_* as shown below. Steric hindrance as well as electrondonating properties of the two phenyl groups prevent hydrazoform.

**Scheme 1:**
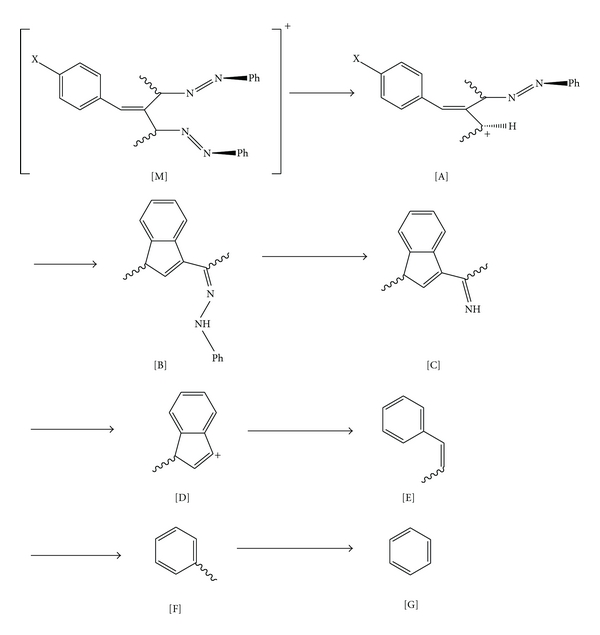
The general route of fragmentation of ligands.

The mass spectroscopic fragmentation pathway of the ligands is shown in Scheme 1. The molecular ions in the mass spectra and their relative abundances are given in Table 4.

Reaction of the ligands  *L*_1-2_, *L*_4-5_, *L*_7–9_, and *L_12_* with Sc(NO_3_)_3_·*x*H_2_O, Y(NO_3_)_3_·6H_2_O, Ce(NO_3_)_3_·6H_2_O, and La(NO_3_)_3_·6H_2_O were performed in ethanol. The complexes show 1 : 1 metal-to-ligand ratio as indicated by their elemental analyses. Their physical properties, magnetic moments, and elemental analyses are listed in Table 5. The results are in agreement with the postulated formulae (±1%). Complexes of ligands 3, 6, 10, and 11 cannot be separated in the solid state.

To achieve an idea about the groups involved in complex formation as well as the influence of the electrical field of the central metal ion on the change distribution within the ligand, the spectra of the complexes were carefully compared with those of the ligands.

The IR spectra of all complexes exhibit broad bands around 3748.80–3413.39 cm^−1^, which are attributed to O–H stretching vibrations of the associated water molecules that may be water of hydration or coordinated molecules. N–H and O–H stretching vibrations of complexes are overlapped for eight complexes. The N–H stretching vibrations of the other ten complexes show shifts to wave numbers differing from those of the free ligand (~315–45 cm^−1^). Red chemical shifts are observed (~7–60 cm^−1^) which are attributed to C=N stretching vibrations. Positive chemical shifts (~10–75 cm^−1^) is also detected for the C=N stretching vibrations and N–H inplane bending vibrations. Red shifts for complexes compared with ligands (~6–35 cm^−1^), (~5–91 cm^−1^), and (~7–50 cm^−1^) are seen for N=N stretching vibrations, N–H inplane bending vibrations, and N–N stretching vibrations, respectively. All these shifts in infrared spectra of the complexes compared with those of the ligands suggest coordination through the two lone pairs of electrons of the two sp^2^ nitrogen atoms of the hydrazoform as a bidentate ligand forming two stable six-membered rings. The infrared spectral data of the complexes are listed in Table 6.

The thermograms of the complexes show a loss of hygroscopic water molecules from 85 to 105°C. The anhydrous complexes show thermal stability up to 130°C. Removal of coordinated water molecules takes place at 130–260°C. The coordinated water molecules are found to be one for complexes Sc-*L*_1_, two for complexes Ce-*L*_1_, Y-*L*_5_, three for complexes Sc-*L*_7_, La-*L*_7_, La-*L*_9_, and Ce-*L*_12_, four for complexes Sc-*L*_9_ and Sc-*L*_12_, and five for complexes Y-*L*_4_, Ce-*L*_4_, Y-*L*_4_, Ce-*L*_9_, and La-*L*_12_. TG shows no coordinated water molecules in the complexes Sc-*L*_2_, Y-*L*_8_, Y-*L*_9_, and Y-*L*_12_.

The magnetic moments of complexes given in Table 7 suggest diamagnetic characters for Sc(III), Y(III), and La(III) complexes whereas Ce(III) complexes have paramagnetic characters ranging from 2.42–2.26 J·T^−1^ [33] being consistent with mononuclear complexes and free from antiferromagnetism. The deviation of the values from the theoretical value suggests that the 4f electron participate in the bond formation of the metal to the ligand.

The thermogravimetric results given in Table 7 and the elemental analyses suggest that Sc-*L_2_* and Y-*L_8_* complexes complete their coordination sphere by ammonia molecules, Y-*L_9_* complete its coordination sphere by ethanol molecules and Sc-*L_1_*, Ce-*L_1_*, Y-*L_4_*, Ce-*L_4_*, Y-*L_5_*, Sc-*L_7_*, La-*L_7_*, La-*L_8_*, Sc-*L_9_*, Ce-*L_9_*, La-*L_9_*, Sc-*L_12_*, La-*L_12_*, and Ce-*L_12_* complexes complete their coordination sphere by water molecules. Y-*L_12_* complex complete its coordination sphere by ammonia and ethanol molecules.

Conductivity measurements using conductivity meter of platinum electrodes for mmol concentrations of complex solutions in *DMF* at 25°C show that Sc-*L_1_*, Ce-*L_1_*, Y-*L_5_*, Y-*L_8_*, Y-*L_9_*, and Y-*L_12_* are neutral, whereas other complexes measure ~213.42–197.63 cm^3^·Ohm^−1^·mol^−1^, suggesting the presence of free nitrate anions.

Elemental analyses, conductivity measurements, magnetic susceptibility measurements, and thermogravimetry of the complexes reinforce each other; suggesting octahedral geometry with coordination number 6 for the complexes Sc-*L_1_*, Sc-*L_2_*, Sc-*L_7_*, La-*L_7_*, Y-*L_8_*, Sc-*L_9_*, and Sc-*L_12_*, whereas they suggest distorted pentagonal bipyramid structure with coordination number 7 for other complexes [34].

**Figure 2:**
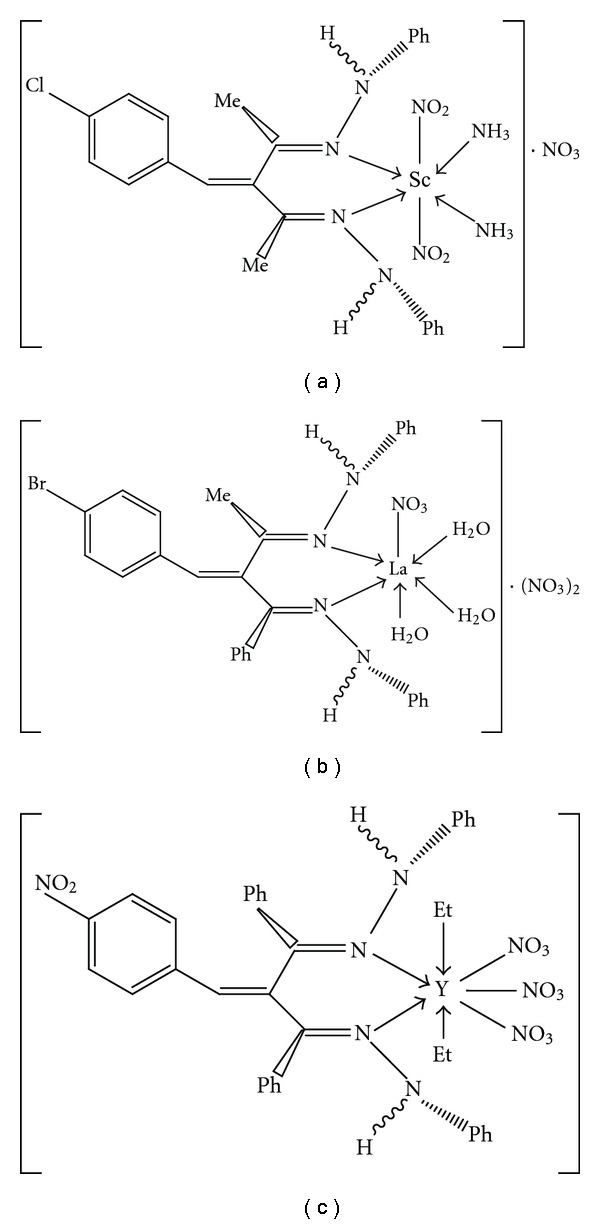
Representative structures of the complexes.

In the light of the above discussion, representative structures of the complexes may be as follow:

The metal complexes and standard drugs (ampicillin, tetracycline, and salicylic acid) were tested for their antimicrobial activity at a concentration of 60 *μ*g mL^−1^ in DMF using the paper disc diffusion method [35, 36]. The diameter of the susceptibility zones was measured, and the results are given in Table 8. The susceptibility zones measured were the clear zones around the discs inhibiting the microbial growth. It is clear that Scandium(III) complexes are more active towards bacterium, yeast, and fungi. Because of the relatively large positive charge density on Scandium atom, it is partially shared with the donor nitrogen atoms of the ligands, and there is *π*-electron delocalization over the whole chelate ring [36, 37]. This in turn, increases its permeation through the lipoid layers of the microorganism membranes. Other factors such as solubility, conductivity, and dipole moment may also increasing activity [36, 37]. Representative structures of the complexes are given in Figure 2.

## 3. Methodology

### 3.1. Chemicals and Equipments

The required 3-benzylidene-2,4-pentanedione, 3-benzylidene-1-phenyl-1,3-butandione, and 3-benzyllidene-1,3-diphenyl-1,3-propanedione were synthesized as described previously [32]. The *Schiff* bases were prepared by condensation with phenylhydrazine (BDH, England) in dry absolute ethanol(Riedel-de Haën) in presence of HCl (Riedel-de Haën) as a catalyst [31]. Sc(NO_3_)_3_·*x*H_2_O 99%, Y(NO_3_)_3_·6H_2_O 99%, Ce(NO_3_)_3_·6H_2_O 99%, and La(NO_3_)_3_·6H_2_O 97% were purchased from BDH. 4-chloro-benzaldehyde 97% (Fluka), 4-fluorobenzaldehyde 98% (BDH), 4-nitrobenzaldehyde 98% (BDH), 4-bromobenzaldehyde 99% (BDH), 1,3-diphenyl-1,3-propandion 98% (BDH), phenyl-1,3-butanedion 99% (BDH), and acetylacetone 99% (WINLAB) were also used. All other solvents used were of the ANALAR grade. Elemental analyses were performed at King Saud University, Saudi Arabia. Melting points were recorded on a Gallenkamp melting point apparatus. IR spectra were recorded on Perkin Elmer (spectrum 1000) FT-IR spectrometer using KBr pellets at the chemistry department, College of Science, King Fahd University for Petroleum and Minerals Saudi Arabia. Proton NMR spectra were recorded using JEOl EX-270 MHz (DMSO-d_6_) with TMS as an internal reference. Mass spectra were recorded with the aid of GCMS-QP 1000 EX Shimadzu spectrophotometer at 70 eV using a direct insertion probe at 25–300°C at the Microanalytical Centre, Cairo University, Egypt. Thermogravimetric analyses were measured under nitrogen flow rate: 30 cm^3^ min^−1^ using a Shimadzu TGA-60H thermobalance from room temperature up to 1000°C at the chemistry department, college of Science, King Faisal University Saudi Arabia. The magnetic susceptibilities were measured using a Sherwood Scientific Ltd. Magnetic susceptibility balance (England).

### 3.2. Preparation of the Ligands

Condensation of substituted {*p*-F(1), *p*-Cl(2), *p*-Br(3), and *p*-NO_2_(4)} 3-benzylidene-2, 4-pentanedione (I), 3-benzylidene-1-phenyl-1,3-butandione (II), and 3-benzyllidene-1,3-diphenyl-1,3-propanedione (III) with phenylhydrazine (see equation below) was performed by refluxing 20 mmol solution of the carbonyl compounds (I_1_ = 4.124, I_2_ = 4.453, I_3_ = 5.342, I_4_ = 4.662 g), (II_1_ = 5.363, II_2_ = 5.690, II_3_ = 6.581, II_4_ = 5.903 g), and (III_1_ = 6.604, III_2_ = 6.932, III_3_ = 7.822, III_4_ = 7.140 g) with 40 mmol phenylhydrazine (3.93 cm^3^) in 30 cm^3^ absolute ethanol in presence of 5 cm^3^ concentrated HCl as a catalyst for 18–24 h. The solution was then cooled to room temperature and added in portions with continuous stirring to crushed ice prepared from bidistilled water. The resulting yield was filtered, washed with water, and recrystallized from ethanol until constant melting point. Color, MP., yield, and elemental analyses are given in Table 1.

### 3.3. General Procedure for the Complexes

A solution containing 5 mmol of ligand in 40 cm^3^ ethanol was refluxed with a solution of 7 mmol of Sc(III), Y(III), La(III), and Ce(III) nitrates for about 12 h after adjusting the pH using ammonia (1 : 1) or Thiel Buffer [38] solution, cooled to room temperature, filtered, washed with ethanol and water, recrystallized from ethanol, and dried on air. The physical properties of the prepared complexes were very stable under ordinary conditions.

### 3.4. Antimicrobial Studies

#### 3.4.1. Preparation of the Discs

The complex (60 **μ**g) in DMF (0.01 cm^3^) was mounted on a paper disc (prepared from blotting paper (5 mm diameter)) with the help of micropipette. The discs were left at room temperature till dryness and then applied on the microorganism-grown agar plates.

#### 3.4.2. Preparation of Agar Plates

Minimal agar was used for the growth of specific microbial species. The preparation of agar plates for *B. subtilis*, *S. aureus*, *E. coli,* and *S. typhi* (bacteria) utilized nutrient agar (2.30 g; obtained from Panreac Quimica SA, Spain) suspended in freshly distilled water (100 cm^3^) and potato dextrose agar medium (3.9 g/100 cm^3^; obtained from Merck) for *A. niger* and *C. albicans* (fungi). This was allowed to soak for 15 min and then boiled on a water bath until the agar was completely dissolved. The mixture was autoclaved for 15 min at 120°C, then poured into previously washed and sterilized Petri dishes, and stored at 30°C for inoculation.

#### 3.4.3. Procedure of Inoculation

Inoculation was done with the help of platinum wire loop, which was heated to red-hot in a flame, cooled and then used for the application of the microbial strains.

#### 3.4.4. Application of the Discs

Sterilized forceps were used for the application of the paper disc on previously inoculated agar plates. When the discs were applied, they were incubated at 37°C for 24 h for bacteria and yeast and at 28°C for 48 h for fungi. The zone of inhibition around the disc was then measured in millimeters [35].

## Figures and Tables

**Table 1:** Physical properties and elemental analyses of the ligands.

	Color	Yield%	MP/°C	Elemental analyses found (calc.)		
				%C	%H	%N
*L_1_*·4H_2_O C_24_H_23_N_4_F 460.46	Yellow	81	213	62.88 (61.65)	6.95 (6.75)	12.23 (12.36)
*L_2_*·7H_2_O C_24_H_23_N_4_Cl 530.8	Yellow	83	238	54.49 (54.40)	7.00 (6.74)	10.60 (11.36)
*L_3_* C_24_H_23_N_4_Br 447.36	Yellow	80	188	64.43 (63.69)	5.14 (500)	12.51 (11.37)
*L_4_* C_24_H_23_N_5_O 397.41	Orange-red	79	163	69.60 (69.73)	5.30 (5.57)	16.50 (16.95)
*L_5_*·3H_2_O C_29_H_23_N_4_F 502	Dark-green	88	225	69.31 (68.87)	6.17 (6.17)	11.15 (10.67)
*L_6_*·3H_2_O C_29_H_25_N_4_Cl 518.88	Light-pink	74	216	67.18 (66.64)	5.97 (5.22)	10.81 (10.41)
*L_7_* C_29_H_23_N_4_Br 509.43	Light-pink	74	20	68.42 (69.13)	4.95 (5.26)	10.99 (10.17)
*L_8_*·3H_2_O C_29_H_25_N_5_O_2_ 530	Orange	67	231	65.78 (65.05)	5.86 (5.25)	13.23 (13.31)
*L_9_* C_34_H_27_N_4_F 510.59	Pale-beige	70	138	80.00 (80.90)	5.32 (5.32)	10.97 (10.95)
*L_10_* C_34_H_27_N_4_Cl 527.04	Beige	88	143	77.48 (76.61)	5.15 (4.82)	10.63 (9.99)
*L_11_* C_34_H_27_N_4_Br 571.47	Beige	65	173	71.46 (71.46)	4.75 (5.31)	9.80 (9.72)
*L_12_* C_34_H_27_N_5_O_2_ 537.58	Orange	70	190	75.00 (74.25)	5.03 (5.22)	13.03 (12.65)

**Table 2:** Selected infrared data of the ligands (cm^−1^).

	**υ**(N–H)	**υ**(C=N)	**υ**(C–N) **δ**(N–H)	**υ**(N=N)	**δ**(N–H)	**υ**(N–N)
*L_1_*	3431	1600	1456	1373	1222	1072
*L_2_*	3430	1598	1443	1376	1249	1071
*L_3_*	3421	1598	1487	1375	1247	1072
*L_4_*	3433	1601	1442	1375	1192	1080
*L_5_*	3447	1598	1502	1365	1226	1098
*L_6_*	3429	1596	1497	1364	1221	1070
*L_7_*	3449	1594	1454	1359	1211	1066
*L_8_*	3346	1597	1444	1346	1240	1060
*L_9_*	3430	1595	1446	1346	1182	1109
*L_10_*	3448	1594	1445	1320	1260	1089
*L_11_*	3441	1595	1446	1359	1251	1072
*L_12_*	3408	1594	1451	1360	1281	1067

**Table 3:** ^1^HNMR spectroscopic data of the ligands (*δ*/ppm).

	2 CH_3_ groups of azoform (d, 3H)	2 CH_3_ groups of hydrazoform (d, 3H)	2 CH groups of azoform (d, 2H)	NH groups of hydrazoform (D_2_O exchangeable, s, 1H)	Aromatic + methylenic protons (m)
*L_1_*	1.588	2.50	3.41	broad	7.12–7.50
*L_2_*	1.61	2.50	3.36	broad	6.85–7.75
*L_3_*	1.60	2.495	3.39	broad	6.70–7.60
*L_4_*	2.00	2.44	3.52	broad	6.71–7.54
*L_5_*	2.24	2.49	3.85	broad	6.56–7.63
*L_6_*	1.37	2.50	4.00	4.80, 7.77	6.91–7.25
*L_7_*	1.436	2.50	3.34	4.83, 7.98	6.83–7.68
*L_8_*	1.45	2.50	3.34	4.79, 8.21	7.06–7.77
*L_9_*	—	—	3.45	—	6.42–7.97
*L_10_*	—	—	3.40	—	6.62–7.95
*L_11_*	—	—	3.50	—	6.50–7.12
*L_12_*	—	—	3.38	—	7.39–8.23

**Table 4:** m/z values (relative intensities) of the main fragments of the ligands.

	[M]^+^	[A]^+^	[B]^+^	[C]^+^	[D]^+^	[E]^+^	[F]^+^	[G]^+^
*L_1_*	386 (11)	281 (20)	263 (19.0)	173 (18.1)	131 (10.40)	118 (31)	92 (12.6)	77 (100)
*L_2_*	402 (0.28)	297 (4.12)	263 (1.01)	173 (6.07)	131 (3.74)	118 (40)	92 (15.54)	77 (100)
*L_3_*	447 (0.12)	342 (1.34)	263 (0.79)	173 (8.90)	131 (2.61)	118 (29.64)	92 (23.06)	77 (100)
*L_4_*	413 (8)	308 (12.7)	263 (41.1)	173 (11.3)	131 (12.7)	118 (28)	92 (53)	77 (100)
*L_5_*	448 (1.11)	343 (6.1)	325 (13.1)	234 (16.2)	131 (4.4)	118 (13.5)	92 (7.7)	77 (97)
*L_6_*	464 (26.0)	359 (22.27)	324 (0.19)	233 (4.64)	131 (0.82)	118 (2.57)	92 (3.67)	77 (100)
*L_7_*	509 18.9	404 —	325 13.1	234 12.5	131 1.0	118 13.5	92 7.7	77 97.3
*L_8_*	475 (8.6)	370 (2.4)	324 (25.9)	233 (4.4)	131 (1.3)	118 (1.0)	92 —	77 (62)
*L_9_*	510 (8)	405 —	386 —	295 (40.2)	193 (11.6)	180 (4.2)	154 (1.1)	77 (72.6)
*L_10_*	527 (3.24)	422 —	387 (2.8)	296 —	194 (12.3)	181 —	155 (2.03)	78 (84)
*L_11_*	571 (7.11)	466 —	386 —	295 32.9	193 8.6	180 25.7	154 —	77 81.4
*L_12_*	537 [M+1]^+^ (10)	432 (8)	386 —	297 (90)	193 (28)	180 (4)	154 (29)	77 (31)

**Table 5:** Physical properties, magnetic moments, and elemental analyses of complexes.

Complex (formula weight)	Color	MP/°C	Yield%	*μ* eff. BM found (calc.)	Elemental analyses found (calc.)		
					%C	%H	%N
Sc[C_24_H_25_N_7_O_10_F] (635.51)	White	255	60	—	46.27 (45.35)	3.70 (3.93)	15.14 (15.43)
Ce[C_24_H_27_N_7_O_11_F] (747.9)	Yellow	302	64	2.31 (2.54)	36.81 (35.50)	3.95 (3.61)	13.11 (13.10)
Sc[C_24_H_29_N_8_O_6_Cl]NO_3_ (667.54)	White	308	72	—	44.06 (43.14)	5.13 (4.34)	18.26 (18.87)
Y[C_24_H_33_N_5_O_7_](NO_3_)_3_·3H_2_O (831.86)	Yellow-orange	288	78	—	35.81 (34.62)	3.87 (4.69)	14.07 (13.46)
Ce[C_24_H_33_N_5_O_7_](NO_3_)_3_·8H_2_O (973.12)	Gray	294	77	2.26 (2.54)	30.81 (29.60)	4.35 (5.04)	12.45 (11.51)
Y[C_29_H_29_N_7_O_11_F] (757.01)	White	313	62	—	45.22 (45.97)	3.74 (3.83)	13.22 (12.95)
Sc[C_29_H_31_N_5_O_6_Br]NO_3_ (731.95)	Yellow	289	54	—	46.92 (44.54)	4.74 (4.23)	11.35 (12.48)
La[C_29_H_31_N_5_O_6_Br](NO_3_)_2_ (887.91)	White	301	81	—	37.92 (39.19)	3.74 (3.49)	10.46 (11.04)
Y[C_29_H_28_N_9_O_11_]·0.5H_2_O (775.95)	Yellow	296	58	—	45.88 (44.85)	4.21 (3.74)	15.99 (16.24)
La[C_29_H_35_N_5_O_7_](NO_3_)_3_ (889.98)	Yellow-white	293	87	—	40.15 (39.10)	4.21 (3.93)	13.42 (12.58)
Sc[C_34_H_35_N_4_O_4_F](NO_3_)_3_·7H_2_O (938.83)	White	314	60	—	42.15 (43.46)	5.31 (5.22)	10.70 (10.44)
Y[C_38_H_39_N_7_O_11_F] (877)	White	287	65	—	52.15 (52.00)	4.85 (4.47)	10.90 (11.18)
La[C_34_H_33_N_6_O_9_F](NO_3_)·4H_2_O (960.85)	Black	299	76	—	43.55 (42.42)	4.99 (4.27)	11.11 (10.20)
Ce[C_34_H_37_N_4_O_5_F](NO_3_)_3_·H_2_O (944)	Yellow-white	301	66	2.42 (2.54)	43.31 (43.22)	4.58 (3.91)	12.21 (10.38)
Sc[C_34_H_35_N_5_O_6_](NO_3_)_3_·H_2_O (857.79)	White	305	79	—	48.22 (47.57)	3.86 (4.38)	(13.05) 14.31
Y[C_38_H_39_N_8_O_13_] (903.69)	White	317	79	—	51.60 (50.44)	4.27 (4.31)	14.04 (12.39)
La[C_34_H_37_N_5_O_7_](NO_3_)_3_·3H_2_O (1011.91)	White	306	63	—	42.20 (43.83)	3.99 (3.22)	10.16 (11.85)
Ce[C_34_H_33_N_6_O_11_](NO_3_)·H_2_O (935.0)	Brown	288	72	2.37 (2.54)	45.27 (43.63)	4.38 (3.52)	10.44 (11.90)

**Table 6:** Selected infrared data of complexes (cm^−1^).

Complexes	**υ**(O–H)	**υ**(N–H)	**υ**(C=N)	**υ**(C–N) + **δ**(N–H)	**υ**(N=N)	**δ**(N–H)	**υ**(N–N)
Sc-*L_1_*	3425	overlap	1598	1503	1365	1225	1079
Ce-*L_1_*	3427	overlap	1596	1499	1397	1248	1069
Sc-*L_2_*	3445	3208	1613	1493	1360	1281	1067
Y-*L_4_*	3445	3300	1610	1503	1381	1227	1097
Ce-*L_4_*	3611	overlap	1648	1457	1372	1228	1157
Y-*L_5_*	3748	overlap	1609	1505	1382	1235	1156
Sc-*L_7_*	3450	3331	1596	1490	1382	1235	1072
La-*L_7_*	3423	overlap	1606	1499	1379	1253	1089
Y-*L_8_*	3455	3395	1597	1512	1345	1224	1062
La-*L_8_*	3538	3342	1594	1511	1382	1246	1105
Sc-*L_9_*	3500	3448	1617	1492	1365	1242	1113
Y-*L_9_*	3540	3116	1592	1444	1361	1210	1168
La-*L_9_*	3523	overlap	1609	1494	1383	1211	1020
Ce-*L_9_*	3413	3115	1591	1489	1356	1208	1168
Sc-*L_12_*	3435	overlap	1594	1491	1360	1290	1067
Y-*L_12_*	3427	overlap	1596	1499	1379	1248	1069
La-*L_12_*	3430	3430	1603	1496	1383	1347	1106
Ce-*L_12_*	3500	3412	1653	1492	1383	1299	1087

**Table 7:** Thermogravimetric results of the complexes.

Complexes	Hygroscopic water				Coordinated water			
	*T*/°C	% Weight loss		No. of water molecule	*T*/°C	% Weight loss		No. of water molecule
		found	calc			found	calc	
Sc[*L_1_*(NO_3_)_3 _(H_2_O)]	—	—	—	—	130–235	2.62	2.78	1
Ce[*L_1_*(NO_3_)_3 _(H_2_O)_2_]	—	—	—	—	Up to 220	4.89	4.72	2
Sc[*L_2_*(NO_3_)_2 _(NH_3_)_2_]NO_3_	—	—	—	—	—	—	—	—
Y[*L_4_*(H_2_O)_5_](NO_3_)_3_·3H_2_O	92	6.18	6.49	3	130–210	9.20	10.82	5
Ce[*L_4_*(H_2_O)_5_](NO_3_)_3_·8H_2_O	90	14.20	12.80	8	130–210	10.20	9.25	5
Y[*L_5_*(NO_3_)_3 _(H_2_O)_2_]	—	—	—	—	160–210	3.99	4.78	2
Sc[*L_7_*(NO_3_)(H_2_O)_3_](NO_3_)_2_	—	—	—	—	170–220	6.42	7.38	3
La[*L_7_*(NO_3_)(H_2_O)_3_](NO_3_)_2_	—	—	—	—	180–240	6.49	6.08	3
Y[*L_8_*(NO_3_)_3 _(NH_3_)]·0.5H_2_O	98	1.13	1.16	0.5	—	—	—	—
La[*L_8_*(H_2_O)_5_](NO_3_)_3_	—	—	—	—	190–250	11.63	10.11	5
Sc[*L_9_*(H_2_O)_4_](NO_3_)_3_·7H_2_O	90	14.60	13.42	7	140–210	6.22	7.67	4
Y[*L_9_*(NO_3_)_3 _(C_2_H_5_OH)_2_]	—	—	—	—	—	—	—	—
La[*L_9_*(NO_3_)_2 _(H_2_O)_3_](NO_3_)·4H_2_O	85	7.00	7.49	4	130–160	5.58	5.62	3
Ce[*L_9_*(H_2_O)_5_](NO_3_)_3_·H_2_O	105	2.47	1.95	1	160–220	8.95	9.73	5
Sc[*L_12_*(H_2_O)_4_](NO_3_)_3_·H_2_O	100	2.05	2.10	1	160–260	9.19	8.39	4
Y[*L_12_*(NO_3_)_3 _(C_2_H_5_OH)_2_]	—	—	—	—	—	—	—	—
La[*L_12_*(H_2_O)_5_](NO_3_)_3_·3H_2_O	95	4.00	5.37	3	130–160	11.70	10.95	5
Ce[L_12_(NO_3_)_2_(H_2_O)_3_](NO_3_)·H_2_O	100	2.17	1.95	1	130–220	5.20	5.86	3

**Table 8:** Antimicrobial activity data for the complexes*.

Complex	*B. subtilis*	*S. aureus*	*E. coli*	*S. typhi*	*A. niger*	*C. albicans*
Sc-*L_1_*	15	7	10	6	+	++
Ce-*L_1_*	9	—	7	—	+	+
Sc-*L_2_*	10	6	6	11	++	++
Y-*L_4_*	7	7	7	7	+	+
Ce-*L_4_*	6	—	7	6	+	++
Y-*L_5_*	10	7	6	8	—	+
Sc-*L_7_*	11	10	10	12	+	+
La-*L_7_*	6	—	—	6	—	—
Y-*L_8_*	6	8	8	8	+	+
La-*L_8_*	6	7	6	6	+	—
Sc-*L_9_*	12	11	12	12	+	+
Y-*L_9_*	14	9	13	9	+	+
La-*L_9_*	6	—	—	—	+	+
Ce-*L_9_*	7	6	6	6	+	+
Sc-*L_12_*	17	10	10	15	++	+++
Y-*L_12_*	11	11	10	8	+	++
La-*L_12_*	11	7	7	—	—	++
Ce-*L_12_*	12	9	10	7	++	+++
Ampicillin	18	16	15	14	—	—
Tetracycline	16	15	17	17	—	—
Salicylic Acid	—	—	—	—	++++	++++

*Inhibition zone diameter (% inhibition): +, 6–9 mm (33–50%); ++, 10–12 mm (55–67%); +++, 13–15 mm (72–83%); ++++, 16–18 mm (89–100%).

Percentage inhibition values were relative to inhibition zone (18 mm) with 100% inhibition.
